# "There's no kind of respect here" A qualitative study of racism and access to maternal health care among Romani women in the Balkans

**DOI:** 10.1186/1475-9276-10-53

**Published:** 2011-11-17

**Authors:** Teresa Janevic, Pooja Sripad, Elizabeth Bradley, Vera Dimitrievska

**Affiliations:** 1 Department of Epidemiology, UMDNJ School of Public Health, 683 Hoes Lane West, PO Box 9, Room 209, Piscataway, NJ 08854, USA; 2 Department of International Health, 615 N. Wolfe Street, Baltimore, MD 21205, USA; 3 Director, Global Health Initiative, New Haven, CT 06520, USA; 4 Population Research Center, University of Groningen, The Netherlands

## Abstract

**Introduction:**

Roma, the largest minority group in Europe, face widespread racism and health disadvantage. Using qualitative data from Serbia and Macedonia, our objective was to develop a conceptual framework showing how three levels of racism--personal, internalized, and institutional--affect access to maternal health care among Romani women.

**Methods:**

Eight focus groups of Romani women aged 14-44 (n = 71), as well as in-depth semi-structured interviews with gynecologists (n = 8) and key informants from NGOs and state institutions (n = 11) were conducted on maternal health care seeking, experiences during care, and perceived health care discrimination. Transcripts were coded, and analyzed using a grounded theory approach. Themes were categorized into domains.

**Results:**

Twenty-two emergent themes identified barriers that reflected how racism affects access to maternal health care. The domains into which the themes were classified were perceptions and interactions with health system, psychological factors, social environment and resources, lack of health system accountability, financial needs, and exclusion from education.

**Conclusions:**

The experiences of Romani women demonstrate psychosocial and structural pathways by which racism and discrimination affect access to prenatal and maternity care. Interventions to address maternal health inequalities should target barriers within all three levels of racism.

## Introduction

Roma, the largest minority group in Eastern Europe, historically have faced widespread racism and discrimination, a position that in many ways has worsened during the post-Communist transition [1]. During transition, Roma faced a period of increased poverty and unemployment, creating an 'underclass' that one sociologist compared with inner-city African Americans in the US [2]. The post-communist era also saw a rise in nationalism and increased hatred toward the Roma, and Roma have been called the most disliked ethnic group in Eastern Europe [1,3]. In Serbia and Macedonia, many Roma face the additional burden of being internally displaced persons (IDPs) or refugees since large numbers, estimated at 80, 000, of Roma fleeing the war in Kosovo were displaced to neighboring regions, including Serbia and Macedonia [4]. To improve the socioeconomic status and social inclusion of Roma, The Decade of Roma Inclusion 2005-2015 was launched in 2005 by the Open Society Institute, World Bank, UNDP, and Roma rights organizations. The project features a commitment to improve the welfare of the Roma in four priority areas, including health, in nine countries that have large Roma populations, including Serbia and Macedonia. Halfway into the decade, progress on access to health care, including maternal health care, remains slow.

Existing data regarding access to maternal health care among Romani women in Serbia and Macedonia, although limited, shows large disparities compared to non-Roma. Eight percent of Romani women in Serbia compared with 1% in the overall population, and 21% in Macedonia compared with 2% in the overall population did not attend any prenatal care visits. Romani women that did attend prenatal care received fewer services, including blood pressure measurement, urine testing, and blood testing [5,6]. In Macedonia, nearly 5% of Romani women in Serbia and 18% had no skilled attendant at birth, compared with 1% and 2% of the general population, respectively. Although we lack data to examine if similar disparities exist in maternal morbidity and mortality, the infant mortality rate among Roma in Serbia was estimated to be 25 per 1000, approximately three times the national average [5].

Despite the growing awareness by international organizations that Roma lack equal access to health care [7], the influence of racism and discrimination on Romani women's access maternal health care has not previously been studied. We sought to elucidate underlying explanations for inequalities in maternal health care, including the role of racism and discrimination. Accordingly, we conducted focus groups with Romani women and interviews with gynecologists and key informants in four cities in Serbia and Macedonia. Our objective was to identify barriers to accessing prenatal and maternity care among Romani women, and to develop a conceptual framework relating racism and discrimination to access to prenatal and maternity care. Serbia and Macedonia are an ideal setting for this research for several reasons. First, Serbia and Macedonia are home to the largest population of Roma in countries not a part of the European Union. Second, these countries both received large numbers of Romani refugees and internally displaced persons from the conflict in Kosovo, a particularly vulnerable group. Finally, while both countries at one time shared the same health system as part of the former Yugoslavia, in Serbia primary health care institutions, which provide prenatal care, and maternity care in hospitals continue to be administered by the State. In contrast, in Macedonia primary health care was privatized in 2006, providing the opportunity for a natural experiment regarding the influence of privatization on accessing prenatal and maternity care among Roma.

We used the terms "race", "racism", and "ethnicity" in this paper as follows. Race is considered a social construct and is used to describe a group of people with similar physical features reflecting ancestry and geographic origins, as identified by others, or as self-identified [8]. Racism is defined as a system of structuring opportunity and assigning value based on the social interpretation of how we look [9]. Ethnicity is used to describe a group that shares cultural and other characteristics [8]. Although "Romani" is most commonly described as an ethnicity, we concur with the argument that utilizing US-based racial theory to examine the situation of the Roma is useful, given their historical social exclusion as a dark-skinned minority group [10]. There also is evidence that the classification "Roma" has features of racial classification, especially when Roma are classified by people of majority groups [11].

## Methods

### Study Design

We conducted the research in June-August 2010 in the capital cities Belgrade, Serbia, and Skopje, Macedonia, and in a small town in each country. The study consisted of a total of eight focus groups with Romani women (n = 71), eight interviews with gynecologists, and 11 interviews with key informants from governmental institutions related to health policy (policy experts) and non-governmental organizations (NGOs) involved in Romani issues (NGO experts). (We are omitting the names of the small towns and organizations to preserve anonymity). We chose a qualitative approach because previous literature was limited and because we sought to elucidate nuanced issues that might link discrimination to utilization of maternity health care. We also wanted to give the opportunity to Romani women to include their voices in the research process. We chose focus groups with Romani women because we felt that the women would feel more comfortable and safe with their peers, and that the interaction between Romani women would stimulate valuable insights. In order to collect information from gynecologists and key informants we used interviews, since given their busy schedules this was the most practical approach for these participants.

We conducted the study following the principles of community-based participatory research [12]. Because Romani women are a marginalized and disadvantaged group, we thought that involving members of the Romani community would enable us to approach the Romani settlements, while at the same time increasing the usefulness of the research to the Romani community. Four Romani women's NGOs participated in the design and choice of questions for the focus group discussion guides, recruitment strategy, recruitment of women, moderation of focus groups, interpretation of results, design of advocacy report, dissemination plan for advocacy report [13]. The NGOs also participated in the design of a pregnancy health brochure for Romani women with a low level of literacy, the idea for which arose from the research results.

### Data Collection

As recommended in qualitative research, we used purposive sampling [14] in which we sought women who had given birth in the past year and were living in Romani settlements. Romani women from NGOs identified women living in Romani settlements and recruited women. The women from the NGOs were familiar to the women of the Romani settlements from their previous work in the settlements, but women from the NGOs did not have personal relationships with the study participants. Therefore the participants felt comfortable with the women from the NGOs and shared the experience of being Romani women, but would not feel coerced to participant or give certain responses due to personal relationships. A recruitment grid was used to ensure diversity in key characteristics including age, literacy, and refugee/internally displaced person status [15]. The size of focus groups ranged from n = 6 to n = 11.

The discussion guide for focus groups included questions on health knowledge and beliefs during pregnancy, what women did after they found out they were pregnant, and experiences during prenatal care and delivery. Questions were reviewed by Romani women's NGOs and adapted to be culturally relevant and comprehensible by women who have no education, i.e. never attended an educational institution. Romani women from the partnering NGOs were trained how to lead focus groups and then moderated all groups, with one moderator and one or two assistants for each group. Women were either the founders of the NGOs or employees chosen by the NGOs based on their availability and interest in the project. The focus group moderators obtained verbal informed consent for participation and for recording of the discussion. The moderators conducted the focus groups in Serbian (n = 2), Romani (n = 2), or Macedonian (n = 4). All groups lasted approximately 90 minutes. The moderator and assistant administered a short survey to collect demographic information. We gave the women a small gift for participation, which they were not aware of during the initial recruitment process. Six of the eight focus groups took place in a community room at the offices of the NGOs, one group took place in the home of a leader of the settlement, and one took place in a community room in the settlement. The Human Subjects Committee of Yale University approved the protocol and study materials.

In addition, we conducted in-depth interviews with eight gynecologists, two in each city, and 11 other key informants. We selected gynecologists using snowball sampling [14,16], and worked in primary health care centers or maternity wards. The first gynecologist, or seed, was identified in each city by NGO contacts or by calling the primary health care center. Interview questions focused on the provider practices, their daily challenges, and experiences with Romani patients. Additional key informants were representatives from NGOs who work with Roma, some of whom were Romani, and policy experts. Interview questions focused on health policy and their perception of barriers to Romani women obtaining prenatal and maternity care. Study participants were identified on transcripts by initials only. Transcripts could not be linked in anyway to personally identifiable information. Only the participating NGOs, principal investigator, and research assistants had access to the transcript files, which were stored on password protected computers.

### Data Analysis

Transcripts were transcribed and translated to English, and then coded and analyzed using the constant comparison method of qualitative data analysis [14,17] to inductively develop emerging themes. All quotes used in the final manuscript were double-checked for agreement on translation by a second translator. Three coders independently coded the transcripts as follows. They developed an initial code structure from transcripts of the first two focus groups and then refined it during review and analysis of transcripts from subsequent focus groups and interviews. Using this final version of the code structure, members of the research team independently coded all transcripts, then discussed discrepancies, achieving consensus and assigning codes to observations by a negotiated process. The code structure was then entered into Atlas.ti, with each code representing one theme. Themes were grouped into domains describing barriers to care. We presented the themes to the participating Romani women's NGOs in order to ensure that they were, from their perspective, consistent with the viewpoint of Romani women.

In the next phase, we placed themes and domains in Jones' conceptual framework regarding three levels of racism and health [18]. This framework was not used during the coding of themes, but rather as a way to organize the themes that had been derived inductively. The first level, personally-mediated racism, is discrimination, which is the individual action of treating a person differently due to their race, based on their prejudice, or differential assumptions regarding someone due to their race. The second, internalized racism, is 'acceptance by stigmatized races of their own abilities and intrinsic worth', resulting in resignation, helplessness, and hopelessness. The third, institutional racism, is defined by Jones as the differential access to goods and opportunities of society by race.

## Results

### Study characteristics

Women ranged in age from 14 to 44 (mean = 25.2, standard deviation = 7.1), and had an average of 2.5 (standard deviation = 1.5) children (Table 1). Approximately a third of the women had no education, and another third had only completed primary school, similar to previously published data describing the education level among Romani women in Serbia [19]. Nearly all were either married or in an unofficial union. Five women were refugees or internally displaced persons from the conflict in Kosovo in 2000.

**Table 1:** Characteristics of study population in focus groups of Romani women, Serbia and Macedonia, n = 71*

Characteristic	n	Mean (sd) or percent
Country		
Serbia	34	50%
Macedonia	34	50%
Residence type		
Capital city	39	57%
Town	29	43%
Age		25.2 (7.1) Range = 14-44
Education		
None	21	33%
Unfinished primary	23	37%
Primary	15	24%
Secondary	3	5%
University	1	2%
Marital status		
Married	41	60%
In union	26	38%
Single	1	1%
		
Number of children		2.5 (1.5) Range = 1-8
Refugee/IDP status		
Yes	5	8%
No	59	92%

* n = 3 women chose not to answer the demographic survey; some column totals do not add to n = 68 due to missing data or to rounding

### Domains and themes

**Figure 1:**
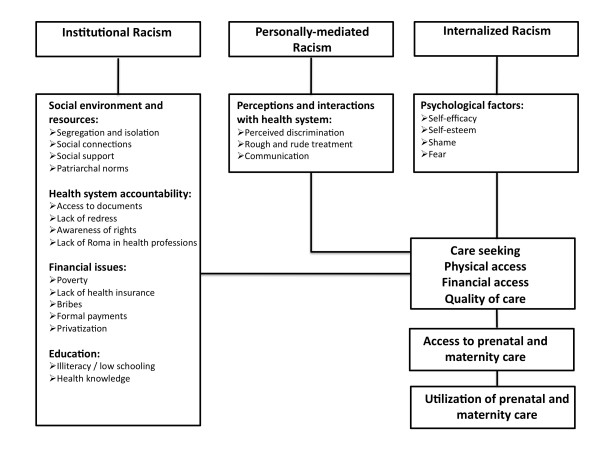
**Three levels of racism and emergent themes from focus groups and interviews regarding access to prenatal and maternity care among Romani women in Serbia and Macedonia, 2010**.

A total of 22 themes emerged during coding and were grouped into six domains (Figure 1). Themes by which personal-level racism affect access to care were grouped in the domain "perceptions and interactions with health providers". The themes in the domain "psychological factors" were conceptualized as consequences of internalized racism. Domains that were conceptualized as a result of institutional racism were "social environment and resources", "health system accountability", "financial issues", and "education". The domains and themes are described in detail below.

#### Personally-mediated racism

##### Perceptions and interaction with health system

The domain "perceptions and interactions with the health system" linked personally-mediated racism to access to maternal health care. Quality of care and patient-provider communication were key issues that arose in discussions. Women described poor treatment, mostly during delivery.

"After the delivery I was not cleaned out well and I had bleeding. They placed me in a separate room alone and nobody came to ask me how I feel, the entire night I bleed till 7 am. I could die.... After this experience I am so afraid to deliver another baby or to become pregnant again, just because of the attitude of the doctors..." (ID01, Romani woman, 37 years, 2 children, Macedonia)

Women also attributed being treated differently because they were Roma.

My doctor is an idiot and I won't go to her anymore...she only yells and shouts. They say that she hates Roma...(ID02, Romani woman, 34 years, 4 children, Serbia)

Women who were refugees from Kosovo also expressed instances of being discriminated against for that reason. For example, one woman said that a nurse told her in the maternity ward, "Why don't you go to Kosovo?" However, often women did not understand and could not articulate the concept of discrimination, despite saying they were treated poorly. This was especially true of women with no education. However, nearly every woman felt that treatment of Romani women is better in Western Europe, as one expressed, "There it is better than here; here there is no sort of respect."

Views on whether Romani were treated differently than other patients varied by type of participant. NGO and policy experts mostly agreed that discrimination does exist toward Roma in the health institutions. Most providers, on the other hand, did not feel that Romani women are treated any differently than other patients,

The way I see it a patient is a patient...I examine them, give them the same treatment as anyone. Any doctor who is a real doctor would do that. (ID03, Gynecologist, Macedonia)

Communication problems were evident, with women stating that they did not understand their doctors, and doctors complaining that Romani women "do not listen" or are "non-compliant".

"The doctor was very good... Only he talked really fast and I couldn't understand him, but my husband understood him and later explained to me." (ID04, Romani woman, 16 years, 2 children, Serbia)

"There are a small percentage who are educated, have one, two, or three children, and come regularly. But those who don't come never come. And they don't listen." (ID05, Gynecologist, Serbia)

Another example of poor communication between Romani women and providers was their interpretation of fear on the part of Romani women. Romani women sometimes said that they were frightened to go the gynecologist, and but one gynecologist mentioned that Romani women may actually come too often to the gynecologist because they have a general "Gypsy fear" in regards to their health. This example highlights complete miscommunication between provider and patient, and inability of the provider to interpret patient behavior. In addition, by attributing a psychological characteristic, fear, to an ethnic group by adding the adjective "Gypsy", the provider revealed how preconceived ideas of the patient based on ethnicity might interfere with the ability to understand the patient's concern for her baby's wellbeing.

Communication problems sometimes reflected cultural differences. When describing why some gynecologists may find it difficult to work with Romani patients, one gynecologist said, "They come in a herd. They come with husband and mother and everyone...and I tell them, what can you see here that you can't see at home?" The desire among Romani women to visit the doctor with family members was not well understood by providers.

#### Internalized racism

##### Psychological factors

Psychological factors that emerged in focus groups and interviews, including lack of self-efficacy, low self-esteem, and fear, seemed to reflect internalized racism and may influence access to maternal health care via care-seeking behaviors. We observed a lack of self-efficacy in the way that Romani women did believe that complaining about their treatment would produce any results. One woman said after saying she had been treated poorly during delivery because she is Roma:

"It was bad, but what could I do? I was in their hands" (ID04, Romani woman, 16 years, 2 children, Serbia)

Romani women also did not appear to think that they had any control of their living conditions:

"But these are our living conditions and we don't have anything better. We try to do the best we can, we try to make it as clean as possible. That's just the way it is." (ID06, Romani woman, 20 years, 3 children, Serbia)

Low self-efficacy also manifested itself as perceived lack of control of their own health.

"... you need to have as a future mother the awareness that your health or your baby's health has priority, and that there are things that you can do to prevent an undesirable result. First that awareness is quite low (among Roma), or rather fatalistic, and (they) think that (they) are pregnant and what will happen will happen." (ID07, NGO expert)

Low self-esteem emerged as a theme in the way that some women felt like they were partially responsible for how they are treated by medical staff:

"But we are also guilty; look at how we go: unwashed, dirty." (ID08, Romani woman, 23 years, 3 children, Serbia)

Then when asked if that is why doctors behave the way they do, the same woman responded,

"We are all poor, but water is free. Maybe because of that (being dirty) they hate us." (ID08, Romani woman, 23 years, 3 children, Serbia)

Shame may result from internalizing discriminatory attitudes, based on gender or Romani ethnicity. One NGO expert mentioned "embarrassment" as a barrier to care seeking, and several women described feeling 'ashamed' during their gynecologist appointment. Some women were also embarrassed to go to a male gynecologist, although others did not mind.

#### Institutional racism

##### Social environment and resources

One domain on the pathway from institutional racism to access to maternal health care was "social environment and resources". Themes in this domain included segregated and isolated poor living conditions, social connections, and patriarchal norms in Romani society. Roma live in highly segregated neighborhoods, often in substandard or illegal housing settlements, and sometimes located on the edges of the city. Women whose families were part of a group of families that had been moved from a more central location to a settlement on the outskirts of the city due to construction of new bridge described the difficulties in getting to the primary health care center,

"I wait for the bus here up to 45 minutes, and you have to catch 2 buses to take your child to the doctor. I'm a single mother with six children and it is not easy for me to leave my children with someone. But if you call an ambulance here you might die." (ID09, Romani woman, 40 years, 6 children, Serbia)

From the perspective of participants who were policy experts, the existence of segregated Romani neighborhoods resulted in Roma being easily overlooked by policy makers. One such participant explained that public health campaigns, such as dissemination of information on free gynecological services, would not likely penetrate Romani settlements, explaining,

"Because you know we live in parallel systems, our neighbors, we don't see them, we cross over each other. We live in different worlds." (ID10, Policy expert, Macedonia)

Women most often were happy to live in communities with other Romani families but were distraught regarding the poor living conditions in which they lived. Women in focus groups complained of living in one room, stench, noise, and a dangerous environment for their children. Worries about how these conditions influence the health of their families superseded concern about going to the doctor.

Discussions among Romani women underscored the importance of social connections. Women perceived that being received by the doctor, waiting times, and quality of treatment all depended on whether they had a "connection" at the primary health care center or hospital. One woman complained that a gynecologist was being rude to her,

"...but then he saw that my mother is a health worker, his behavior changed and he apologized." (ID11, Romani woman, 23 years, 1 child, Serbia)

Romani women, gynecologists, policy experts, and NGO experts remarked on the patriarchal nature of Romani culture, noting it as limiting financial and physical resources for women to use on their health, as well as more direct control of their reproductive health. For example, several women stated that they wanted to have an abortion for their most recent pregnancy but their husband or mother-in-law did not allow them. One NGO expert explained how the woman's place in the household also restricted her access to resources such as money to pay for medical expenses or a cell phone to call to make an appointment.

##### Education

Another domain on the pathway from institutional racism to access to maternal health care was education, which included the themes of lack of schooling and lack of health literacy. Nearly all gynecologists, policy experts, and NGO experts acknowledged that the low level of literacy and health knowledge was a reason that they did not seek prenatal care in a timely fashion or at all. As one gynecologist stated,

"(The education of Roma) needs to be part of the state system...if they don't go to school then they are illiterate." (ID12, Gynecologist, Macedonia)

Some Romani women who said that they did not attend any prenatal care visits did not understand the need to visit the doctor during pregnancy. As one women said,

"I didn't go to the gynecologist during my pregnancy. Why should I go to the doctor? I knew that I was pregnant. I went to the doctor when I felt my contractions." (ID13, Romani woman, 32 years, 5 children, Macedonia)

##### Financial issues

The domain of financial issues also lies on the pathway between institutional racism and access to maternal health care. Themes that fall into the domain of financial issues included poverty, lack of health insurance, informal payments, and privatization of health care. Living in poverty, the invisible opportunity costs of seeking regular prenatal care are particularly high for Romani women. Their first concern is surviving poverty. As the moderator of a focus group described the situation of one of the focus group participants,

"Her youngest recently born daughter was born with Down's Syndrome... she didn't know that she would give birth to a sick child because she didn't go to even one examination before she gave birth. She said that the reason she didn't go is that she couldn't leave the children alone, she didn't have any money, and her husband at that time was in prison." (ID14, Focus group moderator, Serbia)

There appeared to be a gap between policies to enhance Roma access to maternal health care and actual implementation of these policies. In both Serbia and Macedonia, there exist policies to provide pregnant women access to social health insurance, but women are often not connected with these services, largely due to lack of knowledge on the women's side and bureaucracy on the side of the government institutions, particularly in Macedonia. Women may not have a health insurance card due to lack of the necessary personal documents or knowledge of how to obtain one. One NGO expert in Macedonia gave as an example a situation in which an illiterate Romani woman was having difficulties obtaining a health insurance card because she was still registered under her husband's name in a different municipality, and he had to release her from his insurance before she could apply in a new municipality, a process that went on for months in part because she had difficulty understanding the forms and procedures. Many providers do acknowledge that obtaining a health insurance card can sometimes be challenging.

"Pregnant women pay nothing, totally nothing, nothing, nothing. Maybe some medicines that are not on the positive list but are necessary to take during pregnancy they have to pay for.... That means completely free health care. The only problem is with the health insurance cards." (ID15, Gynecologist, Serbia)

Health insurance should pay for all essential services for prenatal care and delivery. However, there is a widespread practice of informal payments, or bribes, in particular for delivery.

The perceived or actual need to pay the doctor a fee or bribe reduced the likelihood of seeking maternal health care. One policy expert explained,

"Some of the Roma women are afraid to go to the hospitals (to give birth), they are afraid that someone will ask them for money." (ID16, Policy expert, Macedonia)

Informal payments, or bribes, were cited as a problem by women in all focus groups. As one woman said,

"They looked for money from me, they didn't want to deliver my baby until my mother-in-law gave them money and then everything was different." (ID17, Romani woman, 18 years, 2 children, Serbia)

Women also perceived that the ability to give informal payments or gifts influences the quality of service provided to patients during delivery and level of negligent behavior shown toward Romani women.

"Next to me was an Albanian woman giving birth, she called the nurse over and gave her a gold bracelet, then the nurse and doctor were the whole time next to her, but they hardly looked at me." (ID18, Romani woman, 20 years, 2 children, Serbia)

The privatization of primary health care in Macedonia emerged as a barrier to Romani women receiving prenatal care due both to the financial barriers it created and to the resulting lack of gynecologists in Romani neighborhoods. At the time of this research, in Shuto Orizari, a large municipality in Skopje in which most of the inhabitants are Roma, there was no gynecologist, and there had rarely been one since privatization. Romani women also reported difficulties finding a doctor to accept them as their primary gynecologist, an administrative requirement to receive some of the free preventive gynecological services. NGO experts and women also described situations when gynecologists charged for services that are supposed to be free or covered by health insurance, so that in effect, informal payments were disguised as formal fees.

##### Health system accountability

Lack of accountability of the health system and the government agencies that oversee the health care delivery and financing system toward inclusion of Roma was another domain on the pathway between institutional racism and maternal health care. Emergent themes included lack of redress, low quality of care, policies prohibiting visitors in maternity wards, lack of awareness of rights on the part of Roma, and lack of Roma in health professions.

Romani women in general did not know how to complain about treatment in health institutions, and key informants expressed that lack of redress was an overall problem with institutions in their countries. One NGO expert grappled with the inability to formally complain in Macedonia when they were made aware that fees were being elicited for Pap smear tests that should be free of charge,

"We phoned the local fund for health insurance that we heard this information, what they answered to us is that no woman has filed an official complaint so they do not have the power to do anything about it. As a NGO we cannot file a complaint, only a woman going through this situation can file a complaint. We are having problems convincing women to file a complaint because the lack of designated gynecologists is pushing them to stay with their designated gynecologist because they have no other option." (ID19, NGO expert, Macedonia)

Low quality of care in state maternity wards was an issue not only for Roma, but overall; in Macedonia, where women also can give birth in private maternity wards, deteriorating quality of care in state maternity wards disproportionately affects Roma and poor women. The failure to ensure quality of care in maternity wards demonstrates lack of accountability on the part of the health system to respond to women's complaints regarding the poor conditions.

"The state hospitals in Macedonia are bad. The black beetles crawl on the babies. There are no basic conditions here. The toilets are awful." (ID20, Romani woman, 35 years, 3 children, Macedonia)

One of the aspects of maternity care about which Romani women were most dissatisfied was that a husband or family member is not allowed to be present during birth, or that in some maternities it is allowed, but only for a fee they said they could not afford. They also complained frequently about being left alone by health care providers. Being left without an advocate during delivery such as a family member, acts to further decrease accountability.

Another issue that policy experts stated influences the care seeking behavior of Romani women is that many do not understand the rights they have to prenatal and maternity care. This was also evidenced in the course of focus groups, when Romani women would ask moderators regarding their rights to social assistance. Last, another emergent theme related to accountability of the health care system is the lack of Roma in health professions. One Romani activist explained,

"Maybe when one day there are more Roma working in the gynecology office, the hospital, etc, then there would be some Romani person to turn to if there was some problem." (ID21, NGO Expert, Serbia)

In another example of how the presence of Romani health workers influences the accountability of health institutions, one young Romani woman described how a Romani obstetrician intervened to prevent another from "cutting her" and delivering her baby by cesarean when the Romani doctor did not deem it necessary.

## Discussion

The framework that we have presented contributes to the understanding of how racism and discrimination toward Roma can work through multiple levels to create inequalities in maternal health care. This framework builds on the traditional supply-demand framework of access to health care [20,21] and Anderson's model of healthcare utilization [22] by explicitly drawing connections between racism and barriers to access to maternal health care. In the traditional supply-demand framework, we can arrange barriers to access to care based on if they arise from the institutional side (e.g. lack of gynecologists) or from the patient side (e.g. lack of knowledge to go the gynecologist). In Anderson's model, we can arrange factors into predisposing characteristics, enabling resources, and need to explain utilization of health services. Our framework goes one step further by placing these factors into the social context of racism to explain how barriers are generated toward a marginalized group such as Roma. By making explicit these connections, the complex ways by which inequalities in maternal health care are produced become apparent.

An important form of personally-mediated racism that was highlighted in our findings is health care discrimination. Health care discrimination is defined as the differential allocation or quality of services based on race, ethnicity, socioeconomic status, or health insurance status [23]. Perceived discrimination related to health service use was found to be more common among Roma in Hungary compared with non-Roma (reported by 35% and 4.4%, respectively) in the only study on the topic, although this measure was not analyzed in association with utilization or quality of care [24]. Disparities in perceived health care discrimination, however, have been associated with lower utilization of health care [25-27] and lower perceived quality of care [28,29] in the U.S, and presents an important avenue for future research among Roma.

Our findings contribute several examples to ongoing discussions in the literature on personally-mediated racism and discrimination in health care, including the measurement of perceived discrimination. The findings from our focus groups suggest that perceived discrimination and quality of care are difficult to measure in very marginalized groups, since low expectations seemed to influence their perception of discrimination and quality of care. In our study, some women perceived difficulties in getting in to see a doctor, longer wait times, and poorer care because they are Roma. In particular, women with relatives who worked in Western Europe and had some exposure to a different standard of care seemed to have higher expectations of how they should be treated. Other women, however, seemed to have low expectations for quality of care, and therefore were not dissatisfied with how they were treated. This seemed to be particularly true for the most illiterate women in our sample, suggesting that it is important to measure expectations when measuring perceived discrimination and quality of care in marginalized groups, and that further research is needed on how to best measure these constructs among Roma.

Our findings also contribute to the current research discussion on health care discrimination by demonstrating how racial bias can be both explicit and implicit [30]. In our research, gynecologists did not perceive any bias toward Roma, but it has been shown that providers can hold implicit racial biases that can influence decision-making and behavior [31]. The communication difficulties between Romani patients and the gynecologist that we identified are examples of how implicit bias can influence quality of care and patient adherence [32,33]. For example, providers complained that Romani women don't listen, while some women said that they had difficulties understanding their doctor, demonstrating a gap in perception of the same patient-provider interaction. Such examples strengthen the understanding of discrimination and health care and thus showcase the utility of studying racism, discrimination and health in different global contexts. The evident lack of communication between providers and patients also indicates the need for increased training for health care workers in communication and cultural competence in this region.

We hypothesized in our model several psychological factors are mediators between internalized racism and access to maternal health care, primarily through influencing care-seeking behavior. Previous work has highlighted the importance of identifying psychosocial factors in understanding the role of race/ethnicity in the utilization of care [34]. Our framework adds to previous work by proposing that an antecedent cause of factors such as low self-esteem and low self-efficacy is internalized attitudes and beliefs due to lifetime prejudicial treatment by society, including the health care system [35]. Prejudice and bias on the part of the provider, either explicit or implicit, can illicit a psychological response, and this response can in turn influence utilization [30]. For example, in our study, one woman was ashamed to go to the doctor due to being "dirty", an emotion that can be viewed as an internalized psychological response to the stereotype that Roma are "dirty". This in turn influenced her care-seeking behavior. The bias on the part of the provider relates to the supply-side, while the influence on care-seeking behavior relates to the demand-side. A second example is the lack of self-efficacy. Past knowledge of inability to affect change, either through personal experience or hearing of other women's experiences, perpetuates a sense of lack of self-efficacy. The lack of accountability on the health care system to respond to women's needs relates to the supply-side, while the lack of self-efficacy relates to the demand-side. Understanding of these mechanisms provides a more nuanced understanding of supply and demand-side barriers to access to maternal health care.

A substantial number of barriers were found on the pathway between institutional racism and access to maternal health care, highlighting the importance of structural barriers in the persistence of health care inequalities [36]. Institutional racism manifests itself throughout the structures of society, including the health care system itself [37]. Although we did find evidence of personally-mediated discriminatory behaviors, as discussed below, it is the institutional level where inequities are produced and allowed to flourish. Thus to address equity effectively, it is essential that inequities be "dismantled" at the institutional level [38]. The Decade of Roma Inclusion is an important attempt at dismantling inequities at the institutional level. Our findings of the intermingling of lack of education and poverty with racial discrimination imply that the multi-sectoral approach employed by the "Decade" is appropriate. However, our findings also demonstrate that the health needs of Romani women are urgent and thus need equal focus and attention as the other priority areas of education, housing, and employment. Careful future analysis of the influence of the Decade initiative on access to health care and health outcomes is necessary.

Some of the institutional barriers support previous literature on factors influencing inequities in health care, while others were unique to the context of our study. Examples of those barriers identified in other contexts include segregation, lack of personal documents or health insurance, or education [36,39]. Barriers identified by our research that have been known to influence access to health care but have less frequently been studied in the context of institutional racism and inequalities in health care include informal payments and privatization. Informal payments, or bribes, are a type of corruption in the health care sector that is endemic to many post-communist countries including Serbia and Macedonia and disproportionately affect those living in poverty, such as Roma [40-42]. This study highlights how corruption in the health sector creates inequalities in access to and quality of health care. This study also serves as a case study of how privatization of health care in Macedonia has decreased access to prenatal care of Romani women through increased costs to patients, shortages of gynecologists in Romani neighborhoods, and decreased quality of state hospitals, thus contributing to institutional racism.

Policy implications can be drawn from all three levels of our conceptual framework in efforts to decrease inequalities in access to maternal health care. At the institutional level, policy implications include increasing accountability of state the health care system for vulnerable groups, decreasing illiteracy among Romani women, and housing policies to improve slum living conditions. Because many institutions are still in transition in both Serbia and Macedonia, it is an opportune time to address these structural issues regarding minority rights. At the level of personally-mediated racism, interventions to improve patient-provider communication and cultural competency should be incorporated into provider training. However, along with such individual-level interventions, organizational change at the institutional level is most likely necessary for their success [43]. Finally, psychological barriers to care such as low self-esteem should be targeted through the empowerment of Romani women. The involvement of Romani women's NGOs with many of these activities is essential, including involvement in provider training, gender equality programs, increasing awareness of patient rights, and maternal health education programs. One outcome of this research project, a pregnancy health education brochure designed specifically for illiterate Romani women for use in outreach by Romani women's NGOs, is an example of how the results can be put into practice.

Our study is the first study on access to maternal health care among Romani women in Serbia and Macedonia, and the design had several strengths. First, Romani women were involved in all phases of the research process, including moderating the focus groups. Second, we obtained data from different stakeholders including gynecologists in order to gain various perspectives on the interaction between health care providers and Romani women. Finally, the fact that we gathered data in two countries and in four cities allowed us to draw conclusions that can be generalized throughout the region. A limitation of our conceptual framework is that while it explores racism as a fundamental cause of lack of access to maternal health care, it does not explicitly address the role of gender and class. Romani women in Southeastern Europe are in a position of triple discrimination, due to racist societal attitudes toward Roma, negative societal attitudes towards persons living in poverty, and gender discrimination in their household due to patriarchal familial norms [44-46]. The theoretical perspective of "intersectionality" stresses the importance of examining together the position of gender, class and race in society in relation to health [47,48]. One emergent theme that arose frequently in focus groups and in interviews was that of "patriarchal culture". During data analyses we included this emergent theme in the domain of "social environment", and thus was included in the framework under institutional racism. However, we acknowledge that "institutional racism" is not an antecedent cause of patriarchal culture, and a more nuanced model would explicitly place gender discrimination as a fundamental cause. Class and entrenched poverty was also was a frequent emergent theme. As the theory of intersectionality suggests, these themes comingled in our data such that they are difficult to extricate. Thus future research should explicitly focus on the intersectionality of gender, class, and Romani ethnicity.

A further limitation of our research is that we did not conduct focus groups with non-Romani women, so we could not draw comparisons between the experiences of the two groups. The quality of maternal health care has been an ongoing issue for the overall population in both countries, and thus some of the experiences described by Romani women may to some degree reflect overall low quality of care rather than an experience specific to Romani women; however, the information we collected suggests that the overall strain put on the health care system due to lack of resources may in some ways increase discriminatory treatment of Romani women. For example, it is the lack of resources that in part creates the need for "connections" to access care, a situation that disproportionately impacts Romani women who are less likely to have connections due to their marginalized place in society. Nonetheless, future research might benefit from investigating the experiences of majority groups in these countries as well.

An additional limitation of our study is that we did not design our focus groups to draw comparisons regarding refugee and internally displaced women. Our limited data suggest that these women are the most likely to not attend any prenatal care and to give birth at home. Future research should sample this most vulnerable group in sufficient numbers to draw more concrete conclusions.

## Conclusion

We have identified many barriers faced by Romani women to access maternal health care in the domains of social environment, financial problems, lack of health system accountability, lack of education, health care discrimination, and psychological factors. By these barriers within the framework of racism, we have exemplified how racism at the institutional, personally-mediated, and internalized levels must be addressed to improve maternal health care for Romani women. Interventions to increase access to maternal health care among Romani women are desperately needed, and should consider barriers at each level in their design.

## Competing interests

The authors declare that they have no competing interests.

## Authors' contributions

TJ designed and conducted the study, carried out data analysis, and wrote the majority of the text. PS and EB contributed to the analysis and interpretation of the data. VD contributed to the study design and participated in coordinating and conducting the study. All authors read and approved the final manuscript.
